# The Role of Erythropoietin and Bone Marrow Concentrate in the Treatment of Osteochondral Defects in Mini-Pigs

**DOI:** 10.1371/journal.pone.0092766

**Published:** 2014-03-27

**Authors:** Marcel Betsch, Simon Thelen, Laila Santak, Monika Herten, Pascal Jungbluth, Daniel Miersch, Mohssen Hakimi, Michael Wild

**Affiliations:** 1 Department of Trauma and Hand Surgery, University Hospital Duesseldorf, Duesseldorf, Germany; 2 Department of and Endovascular Surgery, University Hospital Muenster, Muenster, Germany; 3 Department of Trauma and Orthopaedic Surgery, Klinikum Darmstadt, Darmstadt, Germany; University of Minho, Portugal

## Abstract

**Background:**

All available treatment options for osteochondral and chondral defects do not restore hyaline cartilage and are limited to decreasing associated pain, and maintaining or improving joint function. The purpose of this study was to evaluate the potential of erythropoietin (EPO) in combination with bone marrow aspiration concentrate (BMAC) in the treatment of osteochondral defects of mini-pigs.

**Methods:**

14 Goettinger mini-pigs, in which a 6×10 mm osteochondral defect in the medial femoral condyle of both knee joints was created, were randomized into four groups: biphasic scaffold alone, scaffold with EPO, scaffold with BMAC and scaffold in combination with EPO and BMAC. After 26 weeks all animals were euthanized and histological slides were evaluated using a modified ÓDriscoll Score.

**Results:**

In the therapy groups, areas of chondrogenic tissue that contained collagen II were present. Adding EPO (p = 0.245) or BMAC (p = 0.099) alone to the scaffold led to a non-significant increase in the score compared to the control group. However, the combination of EPO and BMAC in the implanted scaffold showed a significant improvement (p = 0.02) in the histological score.

**Conclusion:**

The results of our study show that in mini-pigs, the combination of EPO and BMAC leads to an enhanced osteochondral healing. However, additional research is necessary to further improve the repair tissue and to define the role of MSCs and EPO in cartilage repair.

## Introduction

Surgical attempts to restore articular cartilage lesions have focused on the recruitment of stem cells by puncturing the subchondral bone to allow bone marrow to flow into the lesions (microfracture) and initiate healing [1]. However, the repair tissue is mostly fibrocartilage and is of variable quantity and inferior quality. Therefore, cell-based therapies such as autologous chondrocyte implantation (ACI), have been developed to treat cartilage and osteochondral lesions [2]. However, these techniques require two surgical procedures, one to harvest the cartilage tissue from a non-weight bearing, unaffected area and a second to implant the cells after their expansion *in vitro* [3]. The *in vitro* expansion of chondrocytes is problematic; the harvested chondrocytes are phenotypically modulated upon proliferation, there are high costs associated with the procedure, and the use of fetal bovine serum can be an issue [4], [5]. Therefore, the development of a single-step, simple, autologous, and cost-effective cartilage repair technique is desirable [6]. An optimal procedure would provide cells for chondro- and osteogenesis, growth factors to enhance the matrix synthesis and also a scaffold that could retain the cells within the defect and protect the newly forming neocartilaginous tissue.

Mesenchymal stem cells (MSCs) within bone marrow aspirates might have advantages over chondrocytes for the treatment of cartilage and osteochondral lesions, since they can be obtained autologously in a less invasive procedure and they can differentiate into chondrocytes, adipocytes and osteoblasts, building complex constructs [7]. In various studies, the benefits of using a cocktail of non-expanded mononuclear cells were shown [8]–[10]. Cell based therapy options using intraoperative, one-step procedures with progenitor cells from bone marrow have shown promising results in musculoskeletal tissues [11], [12].

Non-haematopoietic effects of erythropoietin have been described [13], [14] and linked to the tissue-protective receptor for EPO (EPOR), which belongs to the type 1 cytokine superfamily and is a major molecular component of the injury response [15]. In a study by Shiozawa *et al.* in 2010, EPOR was found on MSCs suggesting a role in the proliferation and differentiation of this cell type [16]. Holstein *et al.* were further able to demonstrate enhanced bone healing in a mouse model with the addition of erythropoietin [17]. Chen *et al.* have shown that erythropoietin has a beneficial effect on the migration and proliferation of bone marrow derived MSCs [18]. EPO protein has also been localized in and around growing chondrocytes, suggesting a role of erythropoietin in cartilage healing [18].

Therefore, the hypothesis of our study was that EPO in combination with bone marrow aspiration concentrate (BMAC) would improve the osteochondral repair in an osteochondral mini-pig defect model.

## Materials and Methods

### Animals

We performed a pre hoc power analysis on the basis that the primary outcome was the modified O'Driscoll score. Given a 4-point difference and a standard deviation of 3 points in a paired t test, seven defects per group provided 80% power to detect this difference. Therefore, fourteen female Goettinger mini-pigs (aged 18–30 months, weight 25–35 kg), with a total of 28 osteochondral defects were used to detect the effects of EPO and BMAC. This study was carried out in strict accordance with the recommendations in the Guide for the Care and Use of Laboratory Animals of the National Institutes of Health. The local Animal Care and Use Committee of the Heinrich Heine University and local government of Duesseldorf (permit number: 87-51.04.2010.A140) approved the animal selection, management and the surgery protocol.

### Animal model and surgery

**Figure 1:**
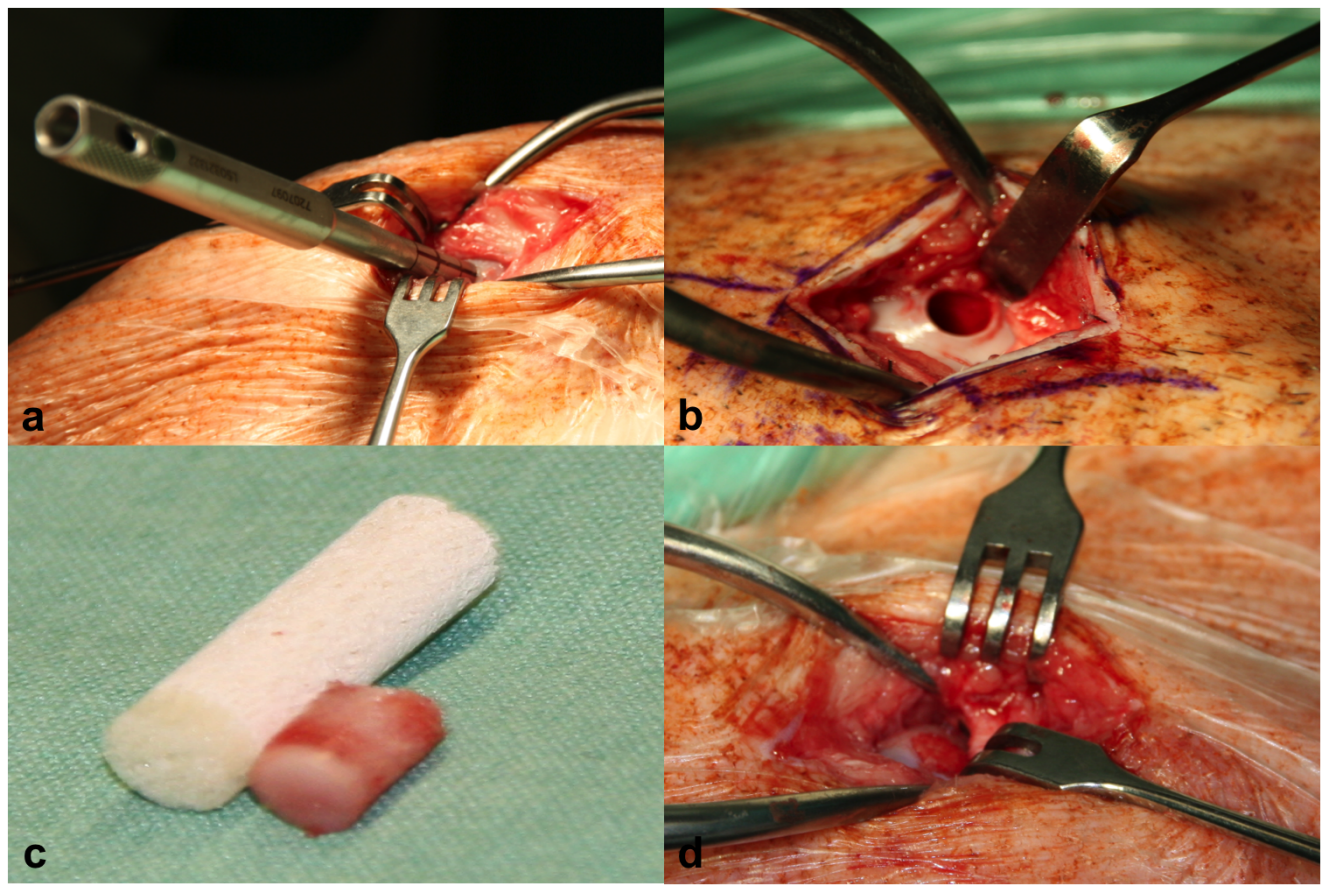
Osteochondral defect. In all 14 mini-pigs, a 6×10 mm cylindrical osteochondral defect in the medial femoral condyle of both knee joints was surgically created with a cylindrical chisel (a). The defect site was exposed and then rinsed with a sterile saline solution (b). An osteochondral cylinder was created with a chisel in comparison to a TRUFIT plug (c). When implanted into the medial femoral condyle it was ensured that the plug was not protruding the surface of the adjacent cartilage (d).

All mini-pigs were acclimated for one week before the surgery, and the defects in all animals were randomized into the following four treatment groups: scaffold alone (control group), scaffold combined with bone marrow aspiration concentrate (BMAC), scaffold combined with erythropoietin (EPO) and scaffold combined with EPO and BMAC (EPO+BMAC) using a sealed envelope system (Table 1). The medial femoral condyles of both knee joints of the 14 animals were used for the experiments, resulting in a total number of 28 defects. In all animals, a 6×10 mm cylindrical osteochondral defect in the medial femoral condyle was surgically created with a cylindrical chisel (Figure 1). The osteochondral defect was filled in all four groups with a biphasic cylindrical scaffold (TRUFIT BGS, Smith & Nephew, USA), which consists of polylactide-co-glycolide, calcium sulfate and polyglycolide fibers. We chose to use this scaffold because of its two layers that simulate cartilage and bony phases, as well as its hydrophilic nature, which can absorb BMAC and/or EPO.

**Table 1:** The defects in all animals were randomized using a sealed envelope system to the following four treatment groups: scaffold alone (control group), scaffold combined with bone marrow aspiration concentrate (BMAC), scaffold combined with erythropoietin (EPO) and scaffold combined with EPO and BMAC (EPO+BMAC).

	Number of Defects	Treatment
**Group 1**	7	scaffold alone (control group)
**Group 2**	7	BMAC + scaffold
**Group 3**	7	EPO + scaffold
**Group 4**	7	EPO + BMAC + scaffold

In the EPO group, the scaffold was soaked with 2 ml of 500 U/ml Erythropoietin alpha (Erypo, Janssen-Cilag GmbH, Neuss, Germany) for 5 minutes, in the BMAC group with 2 ml of BMAC for 5 minutes, and finally in the EPO+BMAC group the scaffold was soaked for 5 minutes with a combination of 1 ml of 500 U/ml Erythropoietin alpha and 1 ml of BMAC prior to implantation. In the control group the defects were filled with the biphasic scaffold alone.

All animals were starved for a minimum of 12 h before surgery. Antibiotic preparation was given perioperatively as a single dose of 3.3 ml Lincomycin (Lincomycin 20%, WdT, Garbsen, Germany). After intramuscular sedation with 0.5 mg/kg Atropin (Atropinsulfat, B Braun, Melsungen, Germany), 5 mg/kg Azaperon (Stresnil, Janssen-Cilag GmbH, Neuss, Germany) and 10 mg/kg Ketamin (Ketavet, Pharmacia GmbH, Karlsruhe, Germany), anesthesia was initiated using 0.5 g Thiopental (Inresa Arzneimittel GmbH, Freiburg, Germany). For all surgical procedures, inhalation anesthesia was performed by use of oxygen and nitrous oxide and isoflurane. Intraoperative analgesia was given by intravenous injection of 0.4 mg/kg Piritramid (Dipidolor, Janssen-Cilag GmbH, Neuss, Germany) and 4.5 mg/kg Carprofene (Rimadyl, Pfitzer Pharma GmbH, Karlsruhe, Germany). For postoperative analgesia, piritramid and carprofene were applied subcutaneously for 3 days. One experienced surgeon performed all surgical procedures in aseptic conditions. The skin of both hind knee joints was scrubbed and disinfected. A midline incision was made in the skin, and the medial condyle exposed through a medial parapatellar approach. A cylindrical osteochondral defect (diameter 6 mm, depth 10 mm) was then created in the medial femoral condyle, with a commercial mosaicplasty 6 mm digging tool. The biphasic osteochondral implant was inserted into the defect with a press-fit technique and care was taken to ensure that the scaffold was flush with the surface of the surrounding articular cartilage. Wound closure was accomplished with bioresorbable sutures and the animals were then returned to their enclosure and monitored until full recovery.

### Bone marrow harvest

Bone marrow (BM) was harvested from the iliac crests of 7 mini-pigs by Jamchidi vacuum aspiration. From the bone marrow aspirate the mononucleated cells were concentrated using a point-of-care device (MarrowStim mini concentration system, Biomet Biologics, Inc., USA). One 30 ml syringe was filled with 6 ml of citrate anticoagulant (ACD-A, Anticoagulant Citrate Dextrose Solution, Biomet Biologics) and 2×12 ml of bone marrow from both iliac crests. This mixture was centrifuged at 600×g for 15 min, and 3–4 ml of BMAC was separated from the cell free plasma/ACD-A mixture and from the red blood cells according to the manufacturers instructions. The concentration of the mononucleated cells in the original BM aspirate and in the BMAC was analysed with an automatic counter (ADVIAs 120, Bayer Diagnostics GmbH, Germany). The concentration factor of the BMAC was calculated from the quotient of mononuclear cells in BM/BMAC.

To evaluate the quality of the aspirated and concentrated bone marrow, the following *in vitro* tests were performed.

### Culture of cells from BM and BMAC

The cells (5.0×10^6^ cells) from BM and from BMAC were cultured in standard cell culture medium DMEM, with 1 g/l glucose, 20% fetal bovine serum, 100 units/ml penicillin, 100 μg/ml streptomycin and 2 mM glutamine (PAA Laboratories GmbH, Austria). Culture conditions were 5% CO_2_ and 37°C.

### CFU assay

The proliferation potency of the mononucleated cells was measured in colony-forming units (CFU), and equal concentrations of mononucleated cells, from BM and from BMAC, were seeded in 24-well plates at cell densities of 1×10^5^ cells/cm^2^. After 7 days of culture the cells were incubated for 60 min at room temperature with hematoxylin to determine the number of CFU.

### FACS analysis

Due to of the limited availability of commercial antibodies cross-reacting with pig antigens only CD14, CD44, CD90 of the recognized MSC-associated markers and CD45 and CD34 (hematopoietic markers) were analyzed by flow cytometry (Cytomics FC 500 FACS; Beckman Coulter, Germany) for the phenotypic characterization of the cultivated cells.

### Postoperative management and sacrifices

All mini-pigs were allowed to fully weight bear and to move freely without any constraints postoperatively. After 26 weeks the animals were euthanized with an overdose of sodium pentobarbital (Eutha 77, Essex Pharma GmbH, Germany). The knee joints were opened and macroscopically evaluated for signs of inflammation, such as tissue reddening, hypertrophy of the villous part of the synovial membrane, tissue adhesions and clarity and colour of the synovial fluid. One specimen from all four experimental groups was randomly selected for cone-beam computed tomography (PaX-Duo3D, Vatech GmbH, Germany). Axial images of 0.08 mm slice thickness were reconstructed using a commercially available DICOM-Viewer (Osirix Imaging Software, Pixmeo) and the reconstructed images were then evaluated for subchondral bone changes. Thereafter, the medial femoral condyles of both knees were excised and fixed in 10% neutral buffered formalin solution for 14 days.

### Staining and Evaluating Specimens

The specimens were dehydrated using ascending grades of alcohol and xylene, then infiltrated, and embedded in methylmethacrylate for non-decalcified sectioning. Any negative influence of heat was avoided by performing controlled polymerization in a cold atmosphere (−4°C). Each specimen was cut in the sagittal direction using a diamond wire saw (Exakt, Apparatebau, Germany). Serial sections were prepared from the central parts of the defect areas, resulting in sections each of approximately 320 μm thickness [19], [20]. All specimens were glued with acrylic cement (Technovit 7210 VLC, Heraeus Kulzer) to the oversize plastic slides (size: 50×100×2 mm, Dia-Plus, Walter Messner GmbH, Germany) and ground to a final thickness of approximately 60 μm. The sections were stained with toluidine blue and safranin-o to evaluate the sulphated glycosaminoglycan (GAG) content and for histological examination. Furthermore, primary mouse monoclonal antibody for collagen II (1∶400, Acris Antibodies GmbH, Germany) and the corresponding non-specific antibody (mouse IgG1) as a negative control were used to stain for collagen II on replicate sections. We used a modified O'Driscoll score (Table 2) for histological evaluation [21]. Two independent experts double-blinded to the groups and treatments, who were familiar with the histology of cartilage repair, evaluated the defects. All scores used for calculating differences between groups were the means of the two independent evaluations.

**Table 2:** Modified ÓDriscoll score.

	Score
**Nature of the predominant tissue**	
***Cellular morphology***	
Hyaline articular cartilage	4
Incompletely differentiated mesenchyme	2
Fibrous tissue	0
***Safranin-O staining of the matrix***	
Normal or nearly normal	3
Moderate	2
Slight	1
None	0
**Structural characteristics**	
***Surface regularity***	
Smooth and intact	3
Superficial horizontal lamination	2
Fissures: 25–100% of the thickness	1
Severe disruption, including fibrillation	0
***Structural integrity***	
Normal	2
Slight disruption, including cysts	1
Severe disintegration	0
***Thickness***	
100% of normal adjacent cartilage	2
50–100% of normal cartilage	1
<50% of normal cartilage	0
***Bonding to the adjacent cartilage***	
Bonded at both ends of graft	2
Bonded at one end, or partially at both ends	1
Not bonded	0
**Absence of cellular changes resulting from degeneration**	
***Hypocellularity***	
Normal cellularity	3
Slight hypocellularity	2
Moderate hypocellularity	1
Severe hypocellularity	0
***Chondrocyte clustering***	
No clusters	2
<25% of the cells	1
5–100% of the cells	0
***Absence of degenerative changes in adjacent cartilage***	
Normal cellularity, no clusters, normal staining	3
Normal cellularity, mild clusters, moderate staining	2
Mild or moderate hypocellularity, slight staining	1
Severe hypocellularity, poor or no staining	0
**Total**	**24**

### Statistical analysis

Normality was evaluated using the Shapiro-Wilk test. We used an unifactorial ANOVA (modified Bonferroni method) to determine differences in the histological score between the experimental groups and for the subgroup analysis of the histological score. Furthermore, we used paired T-tests to check for differences in the white blood cell count and CFUs between the experimental groups. The level of significance was set at p<0.05. Statistical analysis and graphic presentation were prepared using software SPSS 20.0 (SPSS Inc., Chicago, USA).

## Results

### In vitro analysis

**Figure 2:**
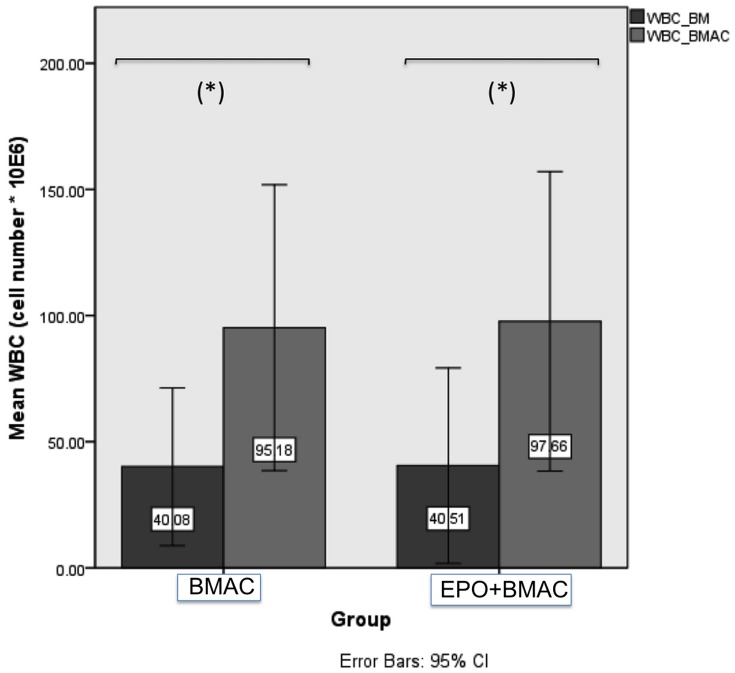
Mean mononuclear cell count. The mean mononuclear cell counts of 7 bone marrow aspirates (BM) compared with 7 bone marrow aspiration concentrates (BMAC) in both groups are shown here (BMAC group and EPO+BMAC group). In both groups, we found a significant increase (*p<0.050*) of the mononuclear cell count in BMAC compared to BM.

The quality of BMAC was evaluated by a calculation of the concentration factor of mononuclear cells in BMAC, the formation of colony forming units, and a cell characterization using FACS analysis of the MSCs within BMAC. The mean value (± standard deviation) of mononuclear cells in bone marrow aspirates of the BMAC group was 40.08×10^6^ cells/ml ± 33.80 and 40.51×10^6^ cells/ml ±41.88 in the EPO+BMAC group, while the mean value of mononuclear cells in the bone marrow aspiration concentrates of the BMAC group was 95.18×10^6^ cells/ml ±61.28 and 97.66×10^6^ cells/ml ±64.20 in the EPO+BMAC group (Figure 2). The resulting concentration factor of the mononuclear cells in the BMAC group was 2.37 (*p = 0.006*) and 2.41 (*p = 0.005*) in the EPO+BMAC group, which is a significant increase of mononuclear cells for both groups that used BMAC. From all animals of the BMAC group adherent cells reached confluence. The CFU were counted in colonies consisting of more than 40 cells with a defined center. After 7 days the mean number of CFU in BM was 1.56±1.91, while in BMAC the mean CFU was 4.83±2.38 (*p = 0.042*). FACS analysis revealed positive staining of CD44 in more than 89.3%±8.0 of all viable single cells. CD14 and CD90 stained 93.6%±9.6 and 93%±13.1 of all viable single cells, respectively. The hematopoietic markers CD45 and CD34 were detected in only 6.4%±2.67 and 2.1%±1.6 of cells.

### Macroscopical appearance

All animals were ambulatory after the surgery, indicating the feasibility of the bilateral defect model. Gross examination of the knee joints revealed that there were no abrasions on the opposing articulating surfaces, and no inflammation of the synovial membrane or other joint tissues was noted. None of the 28 implanted scaffolds appeared dislodged. In no defect did the biphasic scaffold sit above the native cartilage, but two defects in the control group had central depressions. Macroscopically the newly formed tissue appeared to be well integrated with the native cartilage in the therapy groups.

### Histology

**Figure 3:**
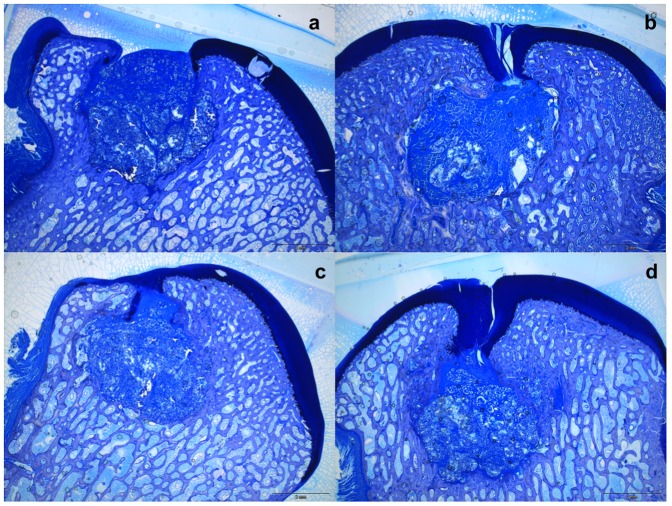
Representative histological slides. Representative histological slides of the medial femoral condyle (toluidine-blue staining) of each group, a) control group b) BMAC c) EPO d) EPO+BMAC. Histologically, residual biomaterials of the biphasic scaffolds were consistently present in the osseous phase of every defect after 26 weeks. In the treatment groups the repair tissue in the cartilage phase stained positive for GAGs.

**Figure 4:**
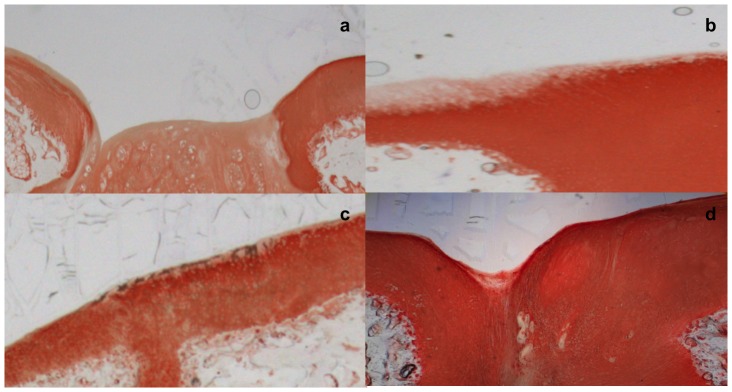
Immunohistochemical analysis for collagen II. Immunohistochemical analysis for collagen II of the regenerate cartilage in representative slides of all four groups. a) control group b) BMAC c) EPO d) EPO+BMAC. In the defects of the therapy groups, areas of chondrogenic tissue, which contained collagen II on the basis of positive immunostaining, were present. However, in the control group the regenerative tissue was generally fibrous. This tissue was deficient in collagen type II as shown by specific staining.

**Figure 5:**
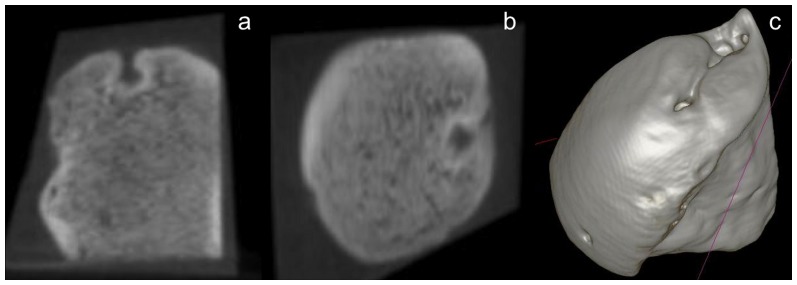
Cone-beam computed tomography. Cone-beam computed tomography of a representative medial femoral condyle delineated a cystic radiolucent area, which was smaller than the originally cylindrical repair area, indicating that the bone adjacent to the implanted scaffold began to remodel starting from the edges of the defect.

In the defects of the therapy groups, areas of chondrogenic tissue were present, as judged from toluidine blue staining and collagen II positive immunostaining, (Figures 3 and 4). However, in the control group the regenerative tissue was generally fibrous. This tissue was deficient in collagen type II immunoreactivity and sGAG, (Figures 3 and 4). Histologically, residual material of the biphasic scaffolds was consistently found in the osseous phase of the defects after 26 weeks. The extracellular matrix of the repair tissue in the bony phase was vascularised but disorganized, with the occurrence of foreign-body giant cells, which indicates that the process of degradation of the scaffold was still on going after 26 weeks. In nine defects, a subchondral bone cyst was noted in the area around the incompletely resorbed scaffold. In these cases cone-beam computed tomography delineated a cystic radiolucent area, which was smaller than the originally cylindrical repair area, indicating that the bone adjacent to the implanted scaffold began to remodel starting from the edges of the defect (Figure 5). However, the normal architecture of the cancellous bone was not restored after 26 weeks.

**Figure 6:**
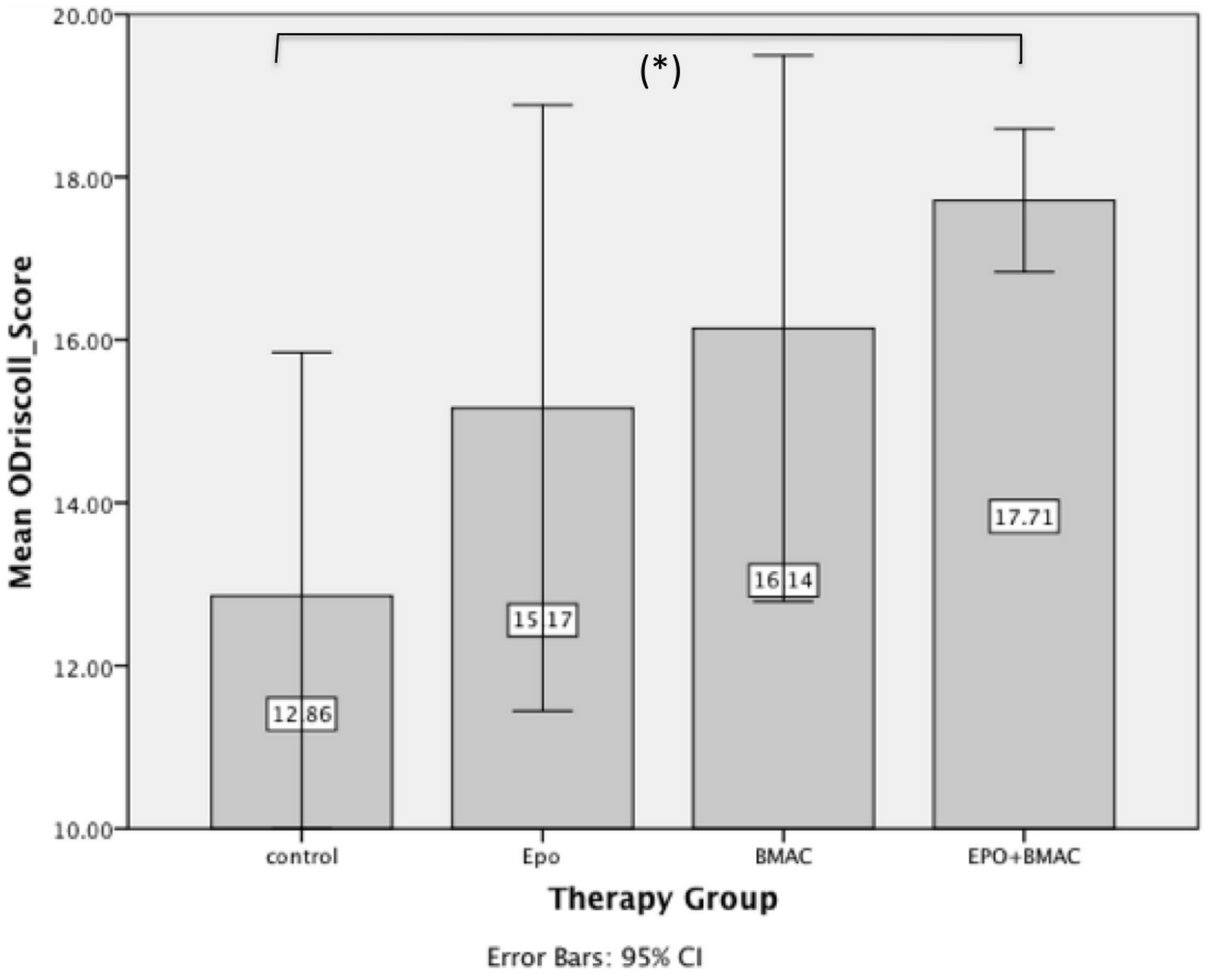
Results modified ÓDriscoll histological scoring. The mean histological score of the four groups. There was a significant increase in the score between the control group and the group with the combination of EPO+BMAC (*p<0.05*).

The lowest mean histological score was for the defects in the control group (12.86±3.24) (Figure 6). Adding erythropoietin (15.17±3.54; EPO group) or BMAC (16.14±5.14; BMAC group) to the scaffold led to a non-significant increase in the score (*p = 0.245 and p = 0.099*). However, the combination of EPO and BMAC (EPO+BMAC group) in the biphasic scaffold led to a significant (*p = 0.020*) increase (17.71±0.95) in the score, compared with the control group (Figure 6). We did not find a significant difference in the scores between the therapy groups of EPO, BMAC and the combination of EPO with BMAC (*p = 0.635 and p = 0.093*). A subgroup analysis of all nine variables in the composite histological score between all groups further revealed a significant difference (*p = 0.047*) only for the variable “thickness” of the regenerated tissue in the EPO versus EPO with BMAC groups.

## Discussion

Despite extensive research efforts, the successful treatment of cartilage and osteochondral defects is still challenging. The results of our study indicate that the combination of EPO and BMAC in a biphasic scaffold can improve osteochondral defect healing significantly as compared with the scaffold alone. We chose the mini-pig as an animal model, because its knee joint resembles the human knee joint and has a 1.5–2.0 mm thick cartilage surface, making it useful for the evaluation of cartilage repair [22]. The implants used have a cartilage phase of polyglycolic acid and poly-D,L-lactide-co-glycolide fibers that are preferentially aligned to provide a porous structural scaffold that allows cellular ingrowth [23]. The bony phase created with calcium-sulfate provides structural stability as well as pores for bony ingrowth. The biphasic design of the scaffold provides cartilage and bone phases with appropriate mechanical properties [24]. This plug is designed to be completely resorbed over time as its being replaced by repair tissue [24]. However, in this study analysis of the histological sections revealed that residual material of the plugs was still present after 26 weeks. The newly-formed tissue in the bony phase had an immature, disorganized structure, containing some cells resembling giant cells accompanied by an infiltration of mononuclear and plasmatic cells. In nine defects, a cyst-like structure, was found at the site of the scaffold, indicating that the cancellous bone adjacent to the implanted material underwent a remodelling process. In a previously published study, CT scans revealed cyst formation instead of bone at the synthetic plug site, suggesting this as a possible healing pattern until the scaffold is completely resorbed [25]. Other literature indicates that this type of scaffold can take up to 2 years to fully resorb, so our finding at 26 weeks is rather short [23], [26]. Rechenberg *et al.* described fibrous tissue and the formation of cyst-like lesions in their study of osteochondral defects, which were most prominent at 6 months, but which disappeared progressively thereafter [27]. However, Jackson *et al.* found extensive resorption of the surrounding bone in their osteochondral defect study in Spanish goats even after 52 weeks [28]. This suggests that in future studies a longer follow-up of up to two years would be necessary to fully evaluate the potential of this scaffold in subchondral bony healing. All defects were filled with the scaffold in press-fit technique. In no defect was the plug dislocated, indicating that this way of implant fixation is secure, even when immediate weight bearing after the surgery is allowed.

The implantation of the biphasic scaffold alone, as used in the control group, led to a mean value of 12.86±3.24 for the overall histological score indicating only a fair result for osteochondral repair. However, the combination of EPO and BMAC as a cell source led to a significant improvement (*p = 0.020*) of the repair with a mean value of 17.71±0.95 for the histological score. Despite this significant improvement, the repair tissue in the cartilage phase was mostly hyaline-like or fibrocartilage. Fibrocartilage may produce good short-term results, but it is less durable than hyaline cartilage leading to unsatisfactory long-term results [29]. It is possible that allowing the animals immediate full weight-bearing after the surgery could have impeded the early cartilage repair and influenced the formation of cartilage, because it is believed that regenerating cartilage should be protected so that the chondrocytes can produce cartilage matrix [28], [30]. In osteochondral defects it also takes time to heal the bone defect, which is necessary to re-establish the subchondral plate [28]. In addition, the cyst formation that was noted after 26 weeks surrounding the incompletely resorbed scaffold could have impeded further improvement of the cartilaginous and osseous healing.

The in vitro expansion of MSCs has various problems, e.g. sterility of the cell culture, high costs, the use of fetal bovine serum and the length of the cultivation, which does require a time-delayed second operation [5]. A promising alternative could be the use of perioperative stem cell concentration, as a single-step procedure by means of density gradient centrifugation of autologous bone marrow [11], [31]. BMAC has been evaluated as a source for MSCs in the regeneration of cartilage and hard tissues in the musculoskeletal system [32], [33]. However, MSCs are only a very small fraction in bone marrow aspirates. Studies suggest that only 0.0001% to 0.001% of mononuclear cells after density gradient centrifugation are MSCs [32], [33]. The number of MSCs can be significantly increased by *in vitro* expansion, but then the technique is no longer a one-step, simple and cost-effective procedure for tissue regeneration. BMAC is also very rich in growth factors like PDGF, TGF-β and VEGF that are thought to be important in joint repair, and the chondro- and osteogenic effect of these growth factors has been shown in multiple studies [34]–[36]. As recommended by Hernigou *et al.* the colony-forming units are used as a quality marker for MSCs [37]. Although CFU is an indicator of the activity of stromal cells, it also indicates the number of transplanted progenitor cells [38]. In this study the mean number of CFU after 7 days was 1.56±1.8 and 4.83±2.38 CFU-F/cm^2^ for BM and BMAC, using a seeding density of 10^5^ cells/cm^2^. These results are comparable with the results of Jäger *et al.* in human cells with an average of 2.8 CFU/cm^2^ for BM and 4.1 CFU/cm^2^ for BMAC [11]. Our results are also in accordance with the results of Castro-Malaspina *et al.* (0.6–1.9 CFU/cm^2^) and Muschler *et al.* (5.5 CFU/cm^2^) [39], [40]. The higher number of CFU from BMAC after 7 days as compared to BM is presumably due the higher number of mesenchymal progenitor cells and the reduction in the number of erythrocytes in the BMAC created by the gradient centrifugation. By using a one-step centrifugation device we were able to concentrate the mononuclear cells by a factor of 2.37 in the BMAC group and 2.41 in the EPO with BMAC group, which is a significant increase (*p = 0.006 and p = 0.005*) in both groups. Our results are lower compared to the 7-fold increase that Thoesen *et al.* achieved in their study with 19 adult dogs, with a different concentration device [41]. Jäger *et al.* and Hermann *et al.* had 5.2 and 4.4 fold increase in the mononuclear cells in human BMAC compared with BM using the same concentration device [11], [42]. The lower concentration of the mononuclear cells in BMAC in our study might be due to the fact that the system we used was developed for use in human BM and species-related differences in cell density might contribute to a lower concentration factor of the BMAC, since the system uses a gradient centrifugation technique to concentrate the BM.

EPO is a 30.4 kD hormone, that has been recognized in recent years as a multifunctional molecule. The anti-inflammatory and anti-apoptotic effects of EPO have been demonstrated in the brain, kidney and heart [43]. EPO docks at the EPOR, which belongs to the cytokine type-1 superfamily, and signalling from that receptor results in inhibition of apoptosis, reduction in inflammation and the mobilization and proliferation of stem cells [15], [16], [18]. These effects prompted us to use EPO in this study to evaluate osteochondral healing. We used a single dose of EPO, which was absorbed into the biphasic scaffold prior to implantation. Previous studies in wound healing and myocardial infarction have shown promising results after a single dose of the here respectively used dosage [44], [45]. In addition, studies have reported that repeated systemic high dose EPO treatments may not further increase the tissue protective effect [17]. The tissue protective effects of EPO require higher concentrations but systemic administration of EPO would also fully activate its haematopoietic and vascular activities, like pro-coagulopathy and haemodynamic effects. To avoid these adverse effects, we chose to put EPO into the scaffold instead of using a systemic application [15]. In 2010, de Spiegelaere *et al.* localized EPO in and around growing cartilage, suggesting a role in cartilage formation and early endochondral ossification [46]. In the present study, the combination of EPO with BMAC led to a significant higher histological score, which can be explained by a positive influence of EPO and BMAC on the local microenvironment; presumably by attenuating inflammation, decreasing necrosis and modulating cell function, as well as recruiting stem cells. The use of EPO alone or of BMAC alone did not significantly enhance the osteochondral healing, suggesting that the combination of a cell source and growth factors is needed to improve osteochondral healing.

A limitation of our study is that the here used biphasic scaffold can take up to two years to fully resorb, therefore it must be considered that at our 6-month evaluation, the repair and resorption process was still ongoing. However, we believe this follow-up is sufficient to provide an indication of the ongoing regenerative process. A further limitation is that all animals were allowed immediate weight-bearing after the surgery, which could have impeded with the early phases of bone and cartilage regeneration. However, weight-bearing restrictions in large animal models are technically challenging and final proof of its efficacy in the cartilage repair of animals is still lacking. In future studies, the use of imaging techniques such as MRI could be useful in addition to a histological score, since these techniques could help to further characterize and quantify the repair tissue in these defects.

### Conclusions

This is the first study that has evaluated the role of EPO in combination with BMAC in an osteochondral defect model. EPO+BMAC leads to an improved osteochondral healing, however additional research is necessary to further improve the quality of the repair tissue and to define the role of EPO in cartilage repair.
